# Gut mucosal colonisation with extended-spectrum beta-lactamase producing Enterobacteriaceae in sub-Saharan Africa: a systematic review and meta-analysis

**DOI:** 10.12688/wellcomeopenres.15514.2

**Published:** 2020-01-24

**Authors:** Joseph M. Lewis, Rebecca Lester, Paul Garner, Nicholas A. Feasey

**Affiliations:** 1 Liverpool School of Tropical Medicine, Liverpool, Merseyside, L3 5QA, UK; 2 Malawi Liverpool Wellcome Clinical Research Programme, Blantyre, Malawi

**Keywords:** ESBL, Extended-spectrum beta-lactamase, Africa south of the Sahara, Antimicrobial resistance

## Abstract

**Background**: Extended-spectrum beta-lactamase producing Enterobacteriaceae (ESBL-E) threaten human health; and, in areas of sub-Saharan Africa (sSA) where carbapenems are not available, may render ESBL-E infections untreatable. Gut mucosal colonisation probably occurs before infection, making prevention of colonisation an attractive target for intervention, but the epidemiology of ESBL-E in sSA is poorly described.

**Objectives**: Describe ESBL-E colonisation prevalence in sSA and risk factors associated with colonisation.

**Methods:** Studies included were prospective cross-sectional or cohort studies reporting gut mucosal ESBL-E colonisation in any population in sSA. We searched PubMed and Scopus on 18 December 2018. We summarise the range of prevalence across sites and tabulated risk factors for colonisation. The protocol was registered (Prospero ID CRD42019123559).

**Results:** From 2975 abstracts we identified 32 studies including a total of 8619 participants from a range of countries and settings. Six studies were longitudinal; no longitudinal studies followed patients beyond hospital discharge.  Prevalence varied between 5 and 84% with a median of 31%, with a relationship to setting: pooled ESBL-E colonisation in community studies was 18% (95% CI 12 to 28, 12 studies); in studies recruiting people at admission to hospital colonisation was 32% (95% CI 24 to 41% 8 studies); and for inpatients, colonisation was 55% (95% CI 49 to 60%, 7 studies). Antimicrobial use was associated with increased risk of ESBL-E colonisation, and protected water sources or water treatment by boiling may reduce risk.

**Conclusions:** ESBL-E colonisation is common in sSA, but how people become carriers and why is not well understood. To inform the design of interventions to interrupt transmission in this setting requires longitudinal, community studies.

## Introduction

Extended-spectrum beta-lactamase producing Enterobacteriaceae (ESBL-E) are a significant threat to human health, and have been identified by the World Health Organisation as pathogens of critical importance ^1^. In sub-Saharan Africa (sSA), it is increasingly clear that a significant proportion of invasive Enterobacteriaceae infections are ESBL-E and the absence of second line antimicrobials can render infections with these pathogens locally untreatable ^2^. Strategies to interrupt ESBL-E transmission that can be practically deployed at scale in low resource settings are urgently needed.

Gut mucosal colonisation with Enterobacteriaceae is thought to precede invasive infection ^3, 4^, and so preventing ESBL-E colonisation is an attractive strategy for prevention of invasive disease. Data describing the basic epidemiology of ESBL-E colonisation in sSA, will help inform the design of interventions targeted at reducing colonisation. A 2016 meta-analysis of community ESBL-E colonisation prevalence among healthy individuals found only four studies from sSA with a pooled prevalence of 15% (95% CI 4–31%), and significant between-study heterogeneity ^5^. No studies described risk factors from Africa. We were aware of a number of studies that had been published since 2016 including a number that described ESBL-E colonisation in any population, so undertook a systematic review and meta-analysis with two aims: firstly, to describe the prevalence of ESBL-E gut mucosal colonisation in sSA; and secondly, to describe any risk factors associated with colonisation. In terms of the PRISMA (preferred reporting items for systematic reviews and meta analyses) PICOS (participants, interventions, comparisons, outcomes and study design) approach, our questions can be framed as: what is the prevalence of ESBL-E gut mucosal colonisation (the outcome) and risk factors for colonisation (comparisons) in any population in sSA (the population) as measured in prospective cross-sectional or cohort studies (study design).

## Methods

Inclusion criteria were any prospective cross-sectional or cohort study that had screened for gut mucosal colonisation of ESBL-E in any population in sSA for which it was possible to extract a numerator and denominator to calculate an ESBL-E colonisation prevalence. Exclusion criteria were studies in which the sampled population was not clearly defined in a reproducible way (i.e. laboratory-based studies), or if the laboratory techniques aimed to isolate only a particular organism or type of organism (e.g. Enteropathogenic *E. coli).* PubMed and Scopus were searched in all fields using the search terms given in Table 1, on 18 December 2018. Abstracts were extracted into Endnote X7.8 (Thomson Reuters, United States) and independently reviewed against the inclusion criteria by two authors (JL and RL), with disagreements settled by consensus. 

**Table 1.** Systematic review search terms.

((ESBL) OR Extended-spectrum beta-lactamase)) AND (((Angola OR Benin OR Botswana OR Burkina Faso OR Burundi OR Cameroon OR Cape Verde OR Central African Republic OR Chad OR Comoros OR Republic of the Congo OR Congo Brazzaville OR Democratic republic of the Congo OR Cote d’Ivoire OR Djibouti OR Equatorial Guinea OR Eritrea OR Ethiopia OR Gabon OR The Gambia OR Ghana OR Guinea OR Guinea-Bissau OR Kenya OR Lesotho OR Liberia OR Madagascar OR Malawi OR Mali OR Mauritania OR Mauritius OR Mozambique OR Namibia OR Niger OR Nigeria OR Reunion OR Rwanda OR Sao Tome and Principe OR Senegal OR Seychelles OR Sierra Leone OR Somalia OR South Africa OR Sudan OR Swaziland OR Eswatini OR Tanzania OR Togo OR Uganda OR Western Sahara OR Zambia OR Zimbabwe) OR Africa))

Full-text review of included studies was then undertaken, with studies assessed against the same inclusion criteria, again with disagreements settled by consensus. Data were then extracted into a Microsoft Excel for Mac v16.27 spreadsheet (Microsoft, United States): study title and authors, year of publication, dates of sample collection, inclusion criteria, median age or participants, details of microbiologic testing procedures, number of participants and number of participants from whom ESBL-E were isolated, and any risk factors for ESBL-E that were assessed and/or found to be associated with ESBL-E colonisation. Two authors extracted data independently (RL and JL) and any inconsistencies corrected by re-review of the original paper. For cohort studies only the baseline prevalence was included. Prevalence was presented as forest plots with exact binomial confidence intervals. Age group (neonate, child, adult, as per study definition) and location of sampling (community, outpatient [including health centre attendees], on hospital admission, [defined as a hospital inpatient for < 24hr] hospitalised, [defined as a hospital inpatient for > 24hr]) were selected as *a priori* subgroups that we hypothesised may explain heterogeneity in ESBL-E prevalence, and analyses were stratified by these subgroups. Studies were additionally classified as being carried out in a *special population* if they were carried out in a subpopulation of a subgroup (for example, pregnant women in the community). Effect size of risk factors for ESBL-E colonisation were presented as odds ratios; if odds ratios were not provided by the original studies then they were calculated, with 0.5 added to zero cells. Pooled random effect summary estimates of prevalence, where calculated, were generated using the *metaprop* package in R using the inverse variance method with a logit transformation. All analysis was undertaken using R v3.5.1 (R Foundation for Statistical Computing, Vienna, Austria).

Risk of bias of included studies was assessed with a modified Critical Appraisal Skills Programme (CASP) checklist, designed to fit our research question (full tool available as *extended data*). The risk of bias assessment was performed by JL and RL, and any disagreements were resolved by consensus.

The protocol of this review was published on PROSPERO (PROSPERO ID CRD42019123559) and the review was undertaken as per Preferred Reporting Items for Systematic Reviews and Meta-Analyses (PRISMA) guidelines (PRISMA checklist available *Reporting guidelines*).

## Results

**Figure 1.**
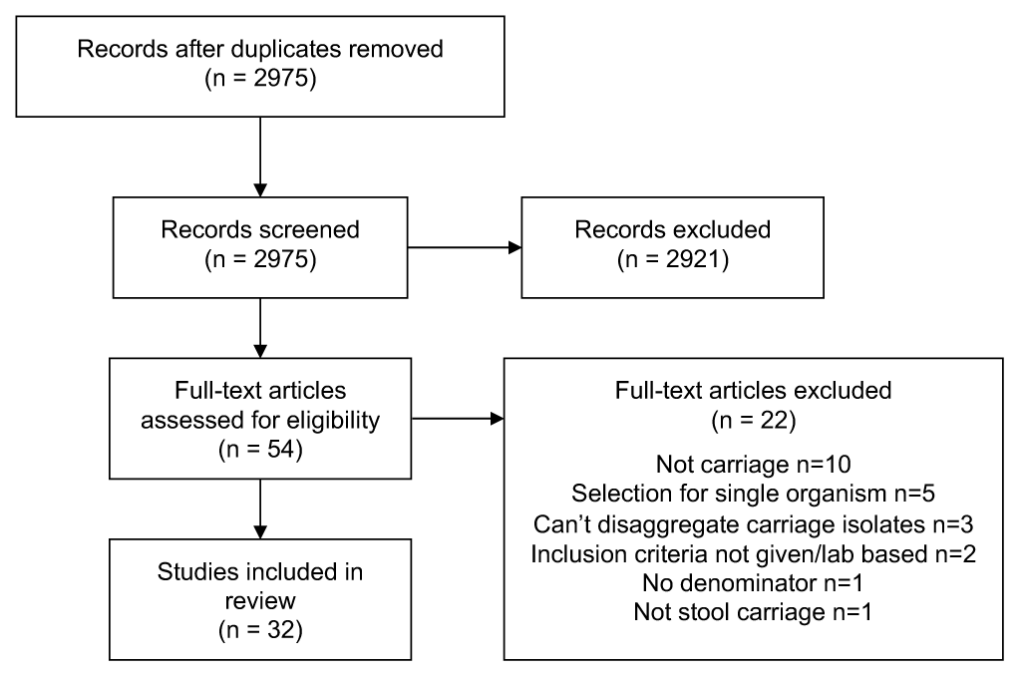
Flow chart of included studies.

Of 2975 identified unique studies, 32 were included in this review ^6– 37^ ( Figure 1), from 19 countries in sSA ( Table 2). Studies from three countries – Tanzania (n=7), Madagascar (n=4) and Cameroon (n=4) - together made up 15/32 (47%) of the available studies. In total, 8619 participants were included and for 7232/8619 (84%) it was possible to disaggregate the participants into age groups: 4313/7232 (60%) were adults, 2470/7232 (34%) children and 449/7232 (6%) neonates. 2302/8619 (27%) of included participants were community members, 1729/8619 (20%) were outpatients, 2836/8619 (33%) were sampled on admission to hospital, and 1534/8619 (18%) were inpatients. 6/32 studies were cohort studies; all of these studies followed patients up whilst hospitalised only. Many studies were carried out in special populations, including the majority of community studies: 9/12 community studies were in special populations, as well as 3/7 outpatient studies, 3/8 studies of participants on hospital admission and 2/7 inpatient studies. It was not possible to classify patients from two studies into our predefined categories: one sampled staff and children of an orphanage, and the other hospital workers and their families. These studies were excluded from the pooled analyses. Details of the microbiological testing procedures are shown in Table 3.

**Table 2.** Details of included studies. CAR = Central African Republic; ART = antiretroviral therapy; UTI = urinary tract infection; NR = not reported. yr = year; m = months, d = days, hr = hours. * = mean rather than media.

Study	Year Pub.	Study Period	Country	Study Type	Inclusion Population: details	Age group	Median age	n
**COMMUNITY STUDIES**								
Albrechtova 2012	2012	2009	Kenya	Cross sec.	General population	Adults	NR	23
Mshana 2016	2016	2014	Tanzania	Cross sec.	General population	both	10yr	334
Katakweba 2018	2018	2011–13	Tanzania	Cross sec.	General population	Adults	NR	70
Ruppe 2009	2009	NR	Senegal	Cross sec.	Special population (remote villages)	Children	6.9yr *	20
Lonchel 2012	2012	2009	Cameroon	Cross sec.	Special population (students)	Adults	24.7yr *	150
Chereau 2015	2015	2013–14	Madagascar	Cross sec.	Special population (pregnant women)	Adults	26yr *	356
Farra 2016	2016	2013	CAR	Cross sec.	Special population (healthy controls in a diarrhoea study)	Children	10.5m	134
Ribeiro 2016	2016	2013	Angola	Cross sec.	Special population (no antibiotics/hospital exposure last 3 mo)	Adults	NR	
Tellevik 2016	2016	2010–11	Tanzania	Cross sec.	Special population: <2yr attending health centre for vaccine	Children	NR	250
Moremi 2017	2017	2015	Tanzania	Cross sec.	Special population (street children)	Children	14.2yr *	107
Chirindze 2018	2018	2016	Mozambique	Cross sec.	Special population (Students in the community)	Adults	NR	275
Sanneh 2018	2018	2015	The Gambia	Cross sec.	Special population (Food handlers in schools)	Adults	37yr *	565
**HOSPITAL OUTPATIENTS**								
Herindrainy 2011	2011	2009	Madagascar	Cross sec.	Outpatients	Adults	NR	306
Lonchel 2012	2012	2009	Cameroon	Cross sec.	Outpatients	Adults	36.9yr *	208
Magoue 2013	2013	2010	Cameroon	Cross sec.	Outpatients	Adults	NR	232
					Outpatients	Children	NR	147
Djuikoue 2016	2016	2011–12	Cameroon	Cross sec.	Special population (outpatient women with susp. UTI)	Adults	NR	86
Wilmore 2017	2017	2014–15	Zimbabwe	Cross sec.	Special population (outpatient, HIV infected, stable on ART)	Children	11yr	175
Herindrainy 2018	2018	2015–16	Madagascar	Cross sec.	Special population (Pregnant women at delivery)	Adults	26yr *	275
Stanley 2018	2018	2017	Uganda	Cross sec.	Special population (participants who reared animals, attending health facility with a fever and/or diarrhoea but without malaria)	both	21.7yr *	300
**ON HOSPITAL ADMISSION**								
Andriatahina 2010	2010	2008	Madagascar	Cohort	On hospital admission	Children	38.3m	244
Kurz 2016	2016	2014	Rwanda	Cohort	On hospital admission	both	29yr	753
Magwenzi 2017	2017	2015	Zimbabwe	Cohort	On hospital admission	Children	1.0yr	164
Founou 2018	2018	2017	South Africa	Cohort	On hospital admission	Adults	NR	43
Moremi 2018	2018	2014–15	Tanzania	Cohort	On hospital admission	Adults	NR	930
Woerther 2011	2011	2007–08	Niger	Cohort	Special population (Children with SAM)	Children	16.3m *	55
Isendahl 2012	2012	2010	Guinea- Bissau	Cross sec.	Special population (Children att. hospital w/ fever or tachycardia)	Children	NR	408
Nelson 2014	2014	2013	Tanzania	Cohort	Special population (Pregnant women and neonates, inpatient)	Neonate	0d	126
						Adults	26.5yr *	113
**INPATIENTS**								
Lonchel 2013	2013	2009	Cameroon	Cross sec.	Inpatients	Adults	46.8yr *	121
Magoue 2013	2013	2010	Cameroon	Cross sec.	Inpatients	Adults	NR	208
Schaumburg 2013	2013	2010–11	Gabon	Cross sec.	Inpatients	Children	NR	200
Desta 2016	2016	2012	Ethiopia	Cross sec.	Inpatients	Adults	35yr	154
					Inpatients	Children	7yr	94
					Inpatients	Neonate	9d	19
Tellevik 2016	2016	2010–11	Tanzania	Cross sec.	Inpatients	Children	NR	353
Nikema Pessinaba 2018	2018	2015–16	Togo	Cross sec.	Special population (<5yr with febrile gastroenteritis)	Children	NR	81
Marando 2018	2018	2016	Tanzania	Cross sec.	Special population (Neonates with sepsis)	Neonate	6d	304
**OTHER**								
Tande 2009	2009	2003	Mali	Cross sec.	Orphanage children	Children	NR	38
					Orphanage staff	Adults	NR	30
Magoue 2013	2013	2010	Cameroon	Cross sec.	Hospital workers and their families	Adults	NR	87
					Relatives and carers of inpatients	Adults	NR	63

**Table 3.** Details of microbiologic testing procedures. NR = not reported; API = analytical profile index; MALDI-TOF = Matrix-Assisted Laser Desorption/Ionization-Time of Flight.

Study	Sample type	Screening method	Speciation method	ESBL confirmation method
Ruppe 2009	Stool	Drigalski and chromagar	NR	Double disc
Tande 2009	Stool	Drigalski with cephalosporin	API	Double disc
Andriatahina 2010	Rectal Swab	Drigalski with cephalosporin	API	Double disc
Herindrainy 2011	Stool	Drigalski with cephalosporin	API	Double disc
Woerther 2011	Stool	Chromagar	API	PCR
Albrechtova 2012	Rectal Swab	Mackonkey with cephalosporin	API	Double disc
Isendahl 2012	Rectal Swab	Chromagar	Vitek	Vitek
Lonchel 2012	Stool	Mackonkey or Drigalski and cephalosporin	MALDI-TOF	Double disc
Lonchel 2013	Stool	Mackonkey or Drigalski and cephalosporin	MALDI-TOF	Double disc
Magoue 2013	Stool	Mackonkey or Drigalski and cephalosporin	NR	Double disc
Schaumburg 2013	Rectal Swab	Chromagar	Vitek	Double disc
Nelson 2014	Rectal Swab	Mackonkey with cephalosporin	Biochemical	Double disc
Chereau 2015	Stool	Drigalski with cephalosporin	API	Double disc
Desta 2016	Stool	Chromagar	Vitek	Vitek
Djuikoue 2016	Stool	Drigalski with cephalosporin	MALDI-TOF	Double disc
Farra 2016	Stool	Chromagar	NR	Double disc
Kurz 2016	Rectal Swab	Chromagar	API	Combination disc
Mshana 2016	Stool	Mackonkey with cephalosporin	API	Chromagar and vitek
Ribeiro 2016	Stool	Chromagar	MALDI-TOF	PCR
Tellevik, 2016	Stool	Chromagar	MALDI-TOF	Combination disc
Magwenzi 2017	Stool or Rectal Swab	Chromagar and Mackonkey with cephalosporin and nutrient broth with cephalosporin	API	Double disc
Moremi 2017	Stool	Mackonkey with cephalosporin	Biochemical	Double disc
Wilmore 2017	Stool	CLEDwith cephalosproin	API and MALDI	Combination disc
Chirindze 2018	Stool	Mackonkey with cephalosporin	API	Double disc
Founou 2018	Rectal Swab	Mackonkey with cephalosporin	API	Combination disc
Herindrainy 2018	Stool or Rectal Swab	Chromagar	MALDI-TOF	Double disc
Katakweba 2018	Stool	Mackonkey with cephalosporin	MALDI-TOF	Double disc
Marando 2018	Rectal swab	Mackonkey with cephalosporin	Biochemical	Double disc
Moremi 2018	Rectal swab	Mackonkey with cephalosporin	vitek	vitek
Nikema Pessinaba 2018	Stool	Drigalski with cephalosporin	NR	NR
Sanneh 2018	Stool	Drigalski And Cephalosporin	NR	Double disc
Stanley 2018	Stool	AST	BD phoenix	BD phoenix

**Figure 2.**
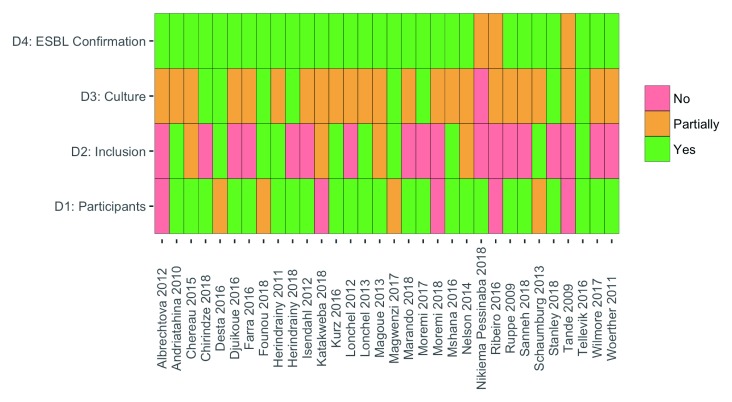
Results of risk of bias assessment. Domain 1: Are the characteristics of the participants included in the study adequately described? Domain 2: Are the eligibility criteria to enter the study explicit and appropriate? Domain 3: Were stool culture results precise and reported? Domain 4: Were the methods of extended-spectrum beta-lactamase (ESBL) confirmatory testing precise?

The results of the risk of bias assessment are shown in Figure 2. The most notable potential for biased ESBL-E prevalence estimates resulted from selection of study populations. Several studies recruited a selected group, which we defined as a special population: pregnant women, street children, children and staff of an orphanage, or food handlers in schools. These are likely to produce a biased estimate of community prevalence. Though microbiological culture methods were frequently described in a reproducible manner, few studies reported quality control procedures, resulting in an assessment of moderate risk of bias for the majority of studies across this domain.

**Figure 3.**
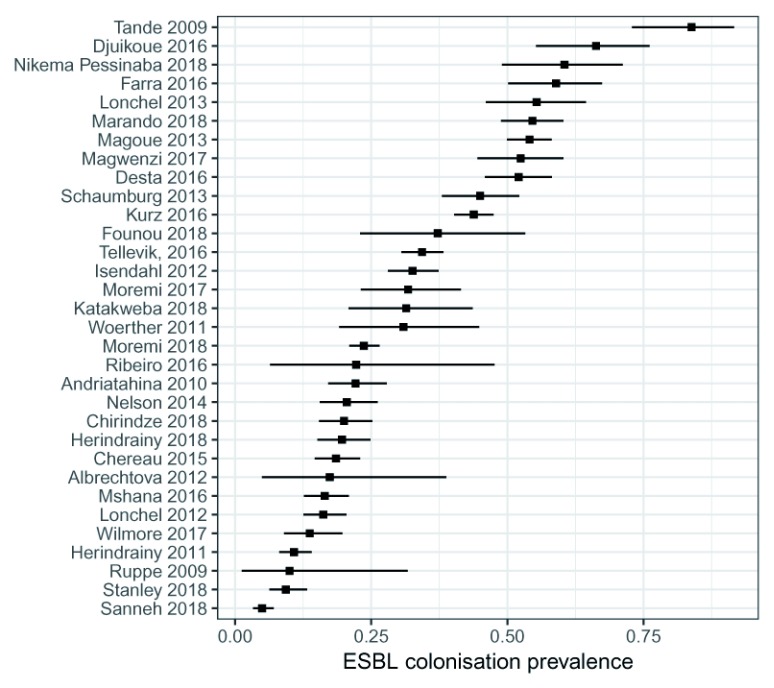
Overall extended-spectrum beta-lactamase producing Enterobacteriaceae (ESBL-E) colonization prevalence by study.

**Figure 4.**
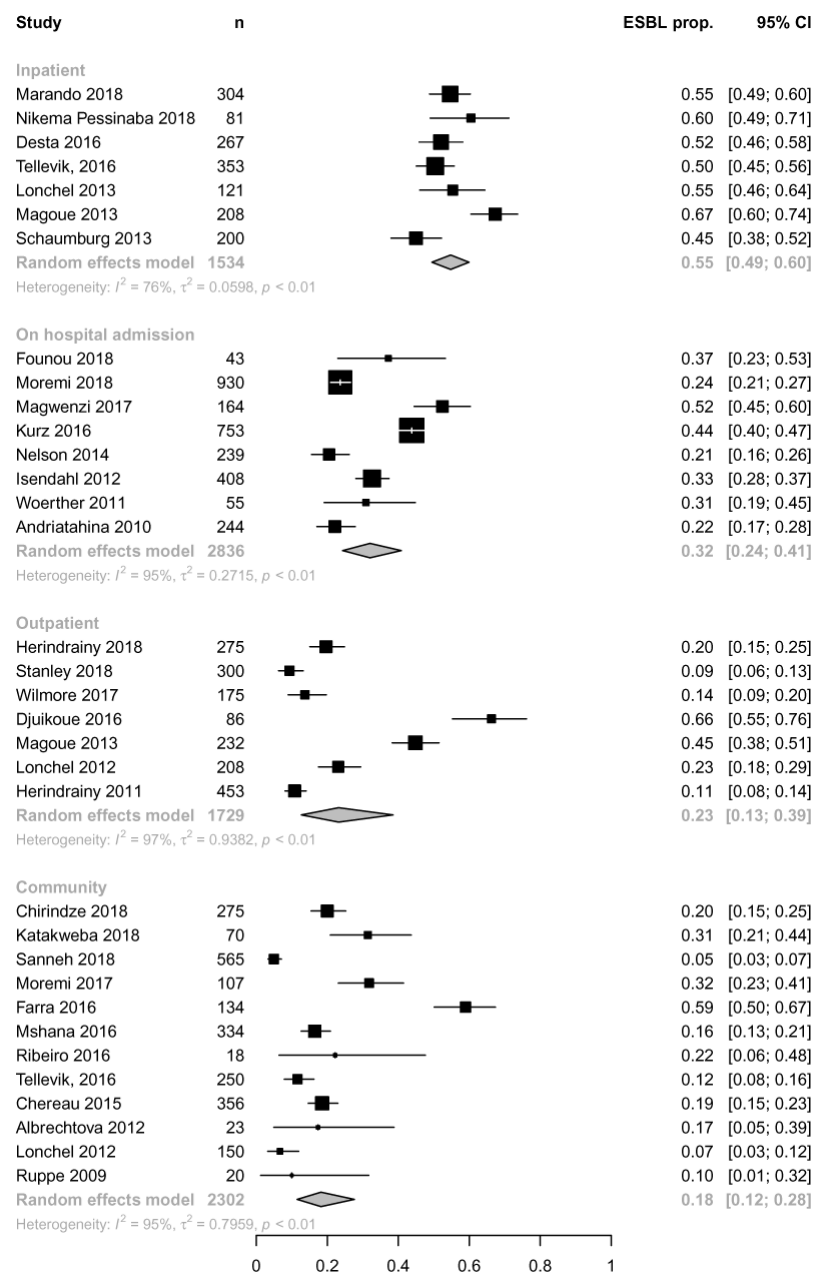
Extended-spectrum beta-lactamase (ESBL) colonisation by study with pooled random effect summary estimates stratified by location of sampling. ESBL prop. = proportion of ESBL producing Enterobacteriaceae.

**Figure 5.**
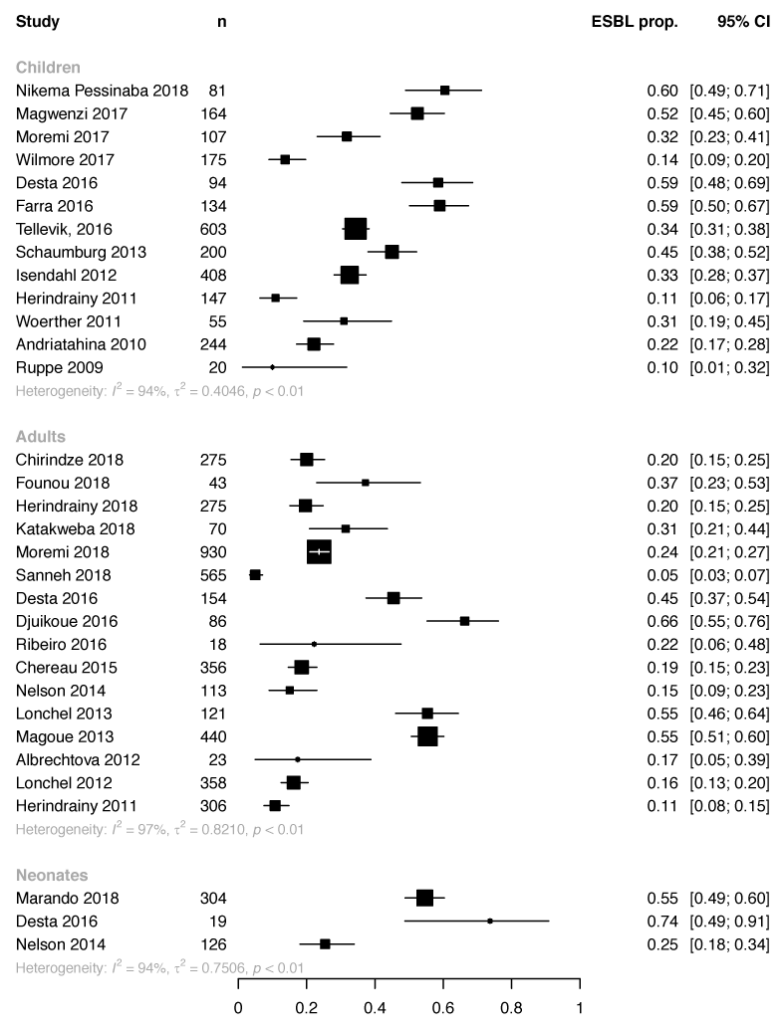
Extended-spectrum beta-lactamase producing Enterobacteriaceae (ESBL-E) carriage prevalence stratified by age group.

Overall ESBL-E colonisation prevalence was extremely heterogeneous across studies ranging from 5–84% (median 31%) with no trend by year of publication ( Figure 3). Some heterogeneity was explained by location of sampling ( Figure 4): inpatients tended to have the highest colonisation prevalence with community members the least. There was no clear difference in prevalence between neonates, children or adults ( Figure 5). Pooled random-effect summary estimates were therefore calculated for differing location of sampling: community members (18% [95% CI 11–28%]), outpatients (23% [95% CI 13-39%]), inpatients on hospital admission (32% [95% CI 24–41%]) and inpatients (55% [95% CI 49-60%]), though in each stratum significant heterogeneity remained (I ^2^ 76–97%) so these summary estimates should be treated with caution ( Figure 4).

Two-thirds (21/32) of studies performed an analysis to identify factors associated with ESBL-E colonisation ( Table 4). Prior hospitalisation was assessed as a risk factor in 13 studies, and a statistically significant association found in 4/13, with odds ratios of 2.1-8.5. Antimicrobial exposure was assessed in 13 studies, and a statistically significant association found in 5/13 with odds ratios of 1.6-27.0. Using water from a borehole ^28^, boiling water before drinking ^14^ and having private inside access to drinking water ^10^ were found to be associated with a lower prevalence of ESBL-E colonisation in three different studies. One study found that a higher socio-economic status was associated with a lower ESBL-E prevalence ^29^, and one the opposite ^13^. Only two studies addressed the association between HIV status and ESBL-E colonisation status; one, in adults found no association ^9^, whereas the other, in children, found a strong association ^17^. Only one study assessed the association between animals in the home as ESBL-E colonisation ^10^, finding no association.

**Table 4.** Assessed and significant risk factors in the included studies. mv = multivariate, uv = univariate, HH = household, abx = antibiotics, SES = socio-economic status, HC = health centre, ART = antiretroviral therapy, VL = viral load, PROM = premature rupture of membranes, WASH = water, sanitation and hygiene. UTI = urinary tract infection, NR = not reported. * confidence interval crosses 1; original publication used fisher’s exact test and found p < 0.05.

Study	Risk factors assessed	Analysis	Significant risk factors	Odds ratio (95% CI)
Tande 2009	Adults with direct contact with the children in orphanage	uv	Contact with orphanage children	19.7 (3.2 - 201.3)
Andriatahina 2010	Age, gender, patient origin (home vs health facility), abx or hospitalisation last 30days, admitting dx, infection on admission	mv	Hospitalisation last 30d	7.4 (2.9-18.3)
Herindrainy 2011	SES, no. of rooms occupied, ratio occupants: room	mv	Occupation HH head unemployed vs manager	9.1 (1.6-53.9)
Isendahl 2012	Age, gender, weight, MUAC, breastfeeding, bedsharing, children in HH, abx, hospitalisation	uv	Bedsharing	1.9 (1.0 - 3.4)
Lonchel 2013	Age, gender, hospital, diagnosis, abx within 3m, hospitalisation within 1yr	mv	Hospitalisation during the previous year	4.13 (1.37–12.78)
			Admission with infection	0.30 (0.10–0.82)
			Intermediate vs tertiary hospital	4.10 (1.77–9.59)
Schaumburg 2013	Age, hospitalisation, residence, sex, diagnosis, abx use	mv	Age <=5	2.2 (1.1–4.8)
			Hospitalization 5–7 days vs < 5	5.1 (1.6–18.4)
			Hospitalization for =7 days vs < 5	30.6 (5.8–566.0)
			Hospital stay during the past 12 months	2.1 (1.1–4.0)
Nelson 2014	For neonates: Gestation, birthweight, gender, delivery method, ward, abx use	uv	Antibiotic use	10.8 (0.6 - 186) *
	For mothers: Delivery mode, admission within 30d, abx within 3m, abx within 30d, current abx, catheter, HIV status		Nothing	
Chereau 2015	Study area, age, education, marital status, type house, electricity, type of birth attendant, toilets, water, animals at home, hospitalisation, abx use	mv	Private inside access to drinking water	0.3 (0.1–0.8)
Desta 2016	Higher maximum bed capacity per room, increasing number of patients admitted in single room	uv	Sharing room vs not	4.0 (2.3 to 5.3)
Djuikoue 2016	Age, pregnancy, abx last 3m, hospital last 3m	uv	None	
Farra 2016	Age, gender, comorbidity, SES, nutritional status, animals at home, toilets, urban/rural, hh members, meals	mv	Highest SES class vs lowest	31.06 (2.49–387.13)
Kurz 2016	Age, gender , residence, ward, referral, other healthcare 3m, abx 3m, education, SES, water source, food, time to HC, caregiver ESBL status	mv	ESBL colonised caregiver,	2.88 (1.80-4.61)
			Antibiotics within 3 months,	2.70 (1.59-4.58)
			Frequently consume eggs	6.52 (1.75-24.31)
			Boil water prior to drinking	0.59 (0.37-0.92)
Mshana 2016	Age, region, no of children in house, abx use within 1m, admission within 1yr	mv	Older age (per yr),	1.07 (1.04–1.10)
			Hospital admission last yr	7.4 (1.43–38.5)
			Abx last 3m	27 (6.63–116),
Tellevik, 2016	Age, gender, residence, parental education, child group, nutritional status, use of abx within 14 days	mv	HIV vs no HIV,	9.99 (2.52–39.57),
			Kinondoni district,	2.62 (1.49–4.60)
			Abx last 14d	1.61 (1.07–2.41)
Moremi 2017	Age, education, herb use, source of income, source of food, street child type	mv	Local herb use,	3.3 (1.31–8.31),
			Sleep on streets vs not	2.8 (1.04–7.65)
Wilmore 2017	Age, gender, CD4, VL, ART duration, admitted to hospital with pneumonia in last 12m, adm to hospital in at 12 m	mv	ART <1yr	8.47 (2.22–2.27)
			Admission with pneumonia in last 12m	8.47 (1.12–64.07)
Marando 2018	Age, gender, weight, admission where, clinical factors, abx use, PROM	mv	Current abx use	1.73 (1.00-2.97),
			ESBL colonised mother	2.19 (1.26-3.79)
Moremi 2018	Age, gender, history of antibiotic use, history of admission, history of surgery	mv	Older age (per year)	1.01 (1.00–1.02)
Nikema Pessinaba 2018	Age, gender, site, drinking water source, time to sample analysis	mv	Drink non borehole water vs borehole	3.47 (1.22-9.82)
Sanneh 2018	WASH behaviours, hospitalised within 3m, invasive procedures, abx within 3m, abx from street, completing abx, diarrhoea/UTI 3m, food handling training	uv	Lack of food handling training and knowledge of the principle of food safety	NR
			Abx within 3m	NR
Stanley 2018	Age, gender, health facility, presentation	uv	none	

Of the 6 cohort studies, all sampled participants on admission to hospital and on discharge, a median 5.6-8 days later, and all found an increase in ESBL-E colonisation prevalence between the two sampling points ( Table 5). No study longitudinally sampled ESBL colonisation in the community, either in community dwellers or in those discharged from hospital.

**Table 5.** Longitudinal ESBL prevalence in included cohort studies. NR = not reported. * = median not given but admission length was 2–10 days.

Study	Study population	ESBL prevalence		Median follow up
		Admission	Discharge	
Andriatahina 2010	Children	51/244 (21%)	88/154 (57%)	5.7d
Woerther 2011	Children	17/55 (31%)	15/16 (94%)	8d
Nelson 2014	Neonates	32/126 (25%)	77/126 (61%)	7d
Kurz 2016	Adults and children	195/392 (50%)	173/208 (83%)	6d
Magwenzi 2017	Children	86/164 (52%)	115/164 (70%)	5.6d
Moremi 2018	Adults	220/930 (24%)	143/272 (53%)	NR *

## Discussion

ESBL-E colonisation is common across sSA, though with significant unexplained heterogeneity between study locations and populations. Community ESBL-E colonisation ranges from 5% in adults in Gambia in 2015 to 59% in children in the Central African Republic in 2013, the latter comparable to the highest described colonisation prevalence in the world ^5^. Our pooled estimate suggests 18% (95% CI 11–29%) of people in sSA are colonised with ESBL-E, a higher prevalence than in high income settings. In Europe, community prevalence of ESBL-E colonisation is reported to range from 3.7% in Spain in 2004 to 7.3% in the UK in 2014 ^38– 41^, similar to the United States where a community prevalence of 3.4% was reported in healthy children ^42^. In many of the estimates of studies included in this review, the reported prevalence of ESBL-E is more comparable to that reported in Asia (46% [95% CI 29–63%] ^5^).

The profound differences in community ESBL-E colonisation prevalence between sSA and high-resource settings warrants further investigation, beyond the assessment of risk factors we have identified in this review. Hospitalisation and antimicrobial use are likely drivers of colonisation in the studies, with higher prevalence seen in hospitalised individuals and prior hospitalisation and antimicrobial exposure frequently identified as risk factors for colonisation. Obversely and consistent with a putative faecal-oral transmission route, use of borehole water, a private indoor water source and boiling water before drinking were associated with reduced ESBL-E colonisation risk, and it may be that poor water, sanitation and hygiene (WASH) infrastructure and practices in sSA are driving high ESBL-E colonisation prevalence. This speaks to a role for poverty in driving ESBL-E colonisation; however, this is likely complex, and context-dependant, as evidenced by conflicting findings of the effect of socio-economic status on colonisation from two studies in different settings.

More broadly, this review highlights areas where data that could inform interventions to interrupt ESBL-E transmission are lacking. In the community, long-term longitudinal ESBL-E colonisation studies are necessary to understand the dynamics of community ESBL-E transmission, particularly the role of within household transmission, and the role of household animals. In health facilities, the determinants of apparent ESBL-E acquisition need to be clearly identified to design pragmatic intervention studies in the context of limited resources. Surprisingly, the role of HIV in driving the high ESBL-E colonisation prevalence in sSA is unknown. HIV is known to profoundly affect gut function, but we identified only two studies which have assessed HIV status as a risk factor for ESBL-E colonisation.

There are limitations of our review. Our search strategy may have missed studies that would otherwise be included. However, using broader inclusion criteria than a recent review of worldwide ESBL-E community colonisation prevalence ^5^, we have identified many more studies from sSA. Risk of bias assessment in observational studies is difficult, with no gold standard, and the tool we have used may misclassify studies with regard to bias. Significant heterogeneity remaining despite stratification warrants caution in interpreting summary estimates. The number of identified studies and participants are small compared to the population of sSA and several countries are over-represented, meaning that care should be taken in generalising these findings across the diverse settings of sSA. Some potentially important risk factors for ESBL-E colonisation (HIV infection and livestock exposure, for example) are not explored in the studies we have identified, and their role in driving colonisation remains unclear.

In conclusion, ESBL-E colonisation in sSA is common, and in places comparable to the highest prevalence in the world, though with significant unexplained heterogeneity between countries and populations. Hospitalisation, antimicrobial use, and poor WASH infrastructure and practices may be contributing to high prevalence; the roles of HIV and animal-human transmission remain unknown. Given the threat to human health of ESBL-E, data to fully characterise routes and drivers of transmission in sSA are necessary to design interventions to interrupt transmission in this setting.

## Data availability

### Underlying data

All data underlying the results are available as part of the article and no additional source data are required.

### Extended data

Zenodo: Risk of bias tool and PRISMA checklist used for the publication: Gut mucosal colonisation with extended-spectrum beta-lactamase producing Enterobacteriaceae in sub-Saharan Africa: a systematic review and meta-analysis, http://doi.org/10.5281/zenodo.3478278 ^43^

This project contains the following extended data:

- Risk of bias tool used in the study

### Reporting guidelines

Zenodo: PRISMA checklist for: Gut mucosal colonisation with extended-spectrum beta-lactamase producing Enterobacteriaceae in sub-Saharan Africa: a systematic review and meta-analysis, http://doi.org/10.5281/zenodo.3478278 ^43^.

Data are available under the terms of the Creative Commons Attribution 4.0 International license (CC-BY 4.0).
