# Supplementary material for: Gut mucosal colonisation with extended-spectrum beta-lactamase producing Enterobacteriaceae in sub-Saharan Africa: a systematic review and meta-analysis
Source: Wellcome Open Res. 2020 Jan 24;4:160. Originally published 2019 Oct 23. [Version 2] doi: 10.12688/wellcomeopenres.15514.2 (PMC6957024; doi:10.12688/wellcomeopenres.15514.2)
Supplement: Peer review history

# Peer review: Reviewer response for version 1

**DOI:** 10.21956/wellcomeopenres.16981.r37453

**Published:** 2020-01-10

**Authors:** Vincent Richard

**Affiliations:** 1 International Department, Institut Pasteur, Paris, France

Among the targets of the AntiMicrobial Resistance, ESBL-producing Enterobacteriaceae are a real worldwide issue not well-studied in Africa. The previous review of Storberg (2014 ^1^) showed ESBL-producing Enterobacteriaceae are a large problem in African healthcare institutions and communities. However, this author highlighted the scarcity of African data about this topic. This new review shows the same trend with only 32 considered studies from 19 countries and among them 15 studies from 3 countries (Tanzania, Madagascar, and Cameroon).

 "Inclusion criteria were any prospective cross-sectional or cohort study that had screened for gut mucosal colonization of ESBL-E in any population in sSA for which it was possible to extract a numerator and denominator to calculate an ESBL-E colonization prevalence." Is that enough to explain that only 32 studies to 2975 were included? These results ask about other countries' lab capacities and about the quality of some works: 2975 identified and 32 included. Does that mean 2943 studies were laboratory-based studies?

 However, the methodology seems to be strong. The risk of bias was included and figure 2 a good way to assess the studies. Could we imagine from this review some proposals for better-implementing studies about this topic, leading to facilitating comparison between countries?

 About prevalence and risk factors the authors seem to be surprised by differences between countries but it is a picture of the high diversity of the Africa region. Once, higher socio-economic status will be a protector because of sanitation and in another country this status will be a risk factor because the load of antimicrobial exposure will be more serious. 

 Because of the design of the studies included in this review, the livestock transmission is not evocated as one of the such risk factors. In discussion, the authors should have to point to the lack of studies concerning assessment of risk from livestock.

 Undoubtedly, the most relevant intervention to reduce the carriage of ESBL-E will be the systematic implementation of WASH infrastructures. However, this kind of intervention will need to be assessed for convincing decision-makers to involve themselves in this strategy.

I confirm that I have read this submission and believe that I have an appropriate level of expertise to confirm that it is of an acceptable scientific standard.

## References

[1] : ESBL-producing Enterobacteriaceae in Africa - a non-systematic literature review of research published 2008-2012. *Infect Ecol Epidemiol*.2014;4: 10.3402/iee.v4.20342 10.3402/iee.v4.20342 24765249PMC3955770

# Response

**Published:** 2020-01-23

**Authors:** Joseph Lewis

**Affiliations:** Liverpool School of Tropical Medicine, UK

We thank the reviewer for their review, and agree that systematic WaSH interventions are likely crucial for tackling antimicrobial resistance in sSA.

2943 studies were excluded at the abstract review stage, largely because they were not relevant to the subject of the review (i.e. were not concerned with ESBL-E carriage at all); others did not take place in sSA, or were review articles, case reports, or studies looking at invasive ESBL-E infection. Unfortunately we did not capture the reasons for rejection for each abstract.

We agree that risk factors for ESBL-E carriage are likely to be context dependent across the diverse settings of sSA. We also agree that there are a number of risk factors (including livestock) that are not explored as potential drivers of ESBL-E carriage in the available studies, and hence their role is not clear. We have amended the limitations section to highlight this.

# Peer review: Reviewer response for version 1

**DOI:** 10.21956/wellcomeopenres.16981.r36859

**Published:** 2019-11-06

**Authors:** Samuel Kariuki

**Affiliations:** 1 Centre for Microbiology Research, Kenya Medical Research Institute (KEMRI), Nairobi, Kenya

Extended spectrum beta-lactamase (ESBL) producing enteric pathogens are a major cause of hospitalization and mortality in sub-Saharan Africa (SSA), more so because alternative options for effective treatment of infections are either too expensive to afford or are completely unavailable in these settings. I believe this review is timely as it provides information on the extent to which data from the region could provide insight into the extent of gut mucosal colonization (a precursor for invasive disease when immune-suppression may happen) and ensure that we institute policies that effectively reduce colonization and control of infections in these settings. 

 The authors observed a rather disturbing trend in data spread across the continent as only a meager 32 studies could qualify to have had data on gut colonization and ESBL testing done. Indeed only 6 of these studies followed up patients beyond the hospital discharge. The authors observed that antimicrobial use was associated with increased risk of ESBL-E colonization, and protected water sources or water treatment by boiling may reduce risk in affected patients.

 The authors did their best to review all available data to answer their key review questions. The methodology was robust and systematic, and the analysis is complex but easy to follow. The major weakness in this review (which is really non-methodological) is the small number of studies available for the large population of SSA and for which major conclusions to be drawn from such a small sample size would be greatly flawed. There is no doubt that gut mucosal colonization with ESBL-producing gut pathogens plays a major role as a risk factor for invasive disease in hospitalized patients, this has been shown in studies in other parts of the world and such evidence is therefore crucial to compare with SSA. The poor implementation of WaSH in communities and Infection Prevention and control (IPC) strategies in healthcare settings certainly add to the challenges associated with prevention of gut-associated mucosal colonization with ESBL-producing bacteria. It is crucial that the authors clearly indicate the major flaw with the conclusion especially as it is based on a rather small and thinly spread number of studies in SSA. Although HIV and the role of livestock transmission of these zoonotic pathogens in studies in SSA was inconclusive, the fact that the studies reviewed may not necessarily have had these as study objectives cannot be ruled out. Interpretation of such review data should be therefore done with caution especially pertaining to possible key risk factors in disease transmission and gut colonization.

I confirm that I have read this submission and believe that I have an appropriate level of expertise to confirm that it is of an acceptable scientific standard.

# Response

**Published:** 2020-01-23

**Authors:** Joseph Lewis

**Affiliations:** Liverpool School of Tropical Medicine, UK

We thank the reviewer for their review, and agree that the available data are scanty and it is not at all clear that the findings are generalisable across the diverse countries of sub-Saharan Africa, especially as some countries are over-represented in the dataset. We have amended the limitations section of our manuscript to highlight the lack of data and, in particular, that the role of animal to human transmission and HIV infection play in driving ESBL-E colonisation in sSA is unclear.
